# Local plant adaptation across a subarctic elevational gradient

**DOI:** 10.1098/rsos.140141

**Published:** 2014-11-12

**Authors:** Paul Kardol, Jonathan R. De Long, David A. Wardle

**Affiliations:** Department of Forest Ecology and Management, Swedish University of Agricultural Sciences, 90183 Umeå, Sweden

**Keywords:** aboveground–belowground linkages, *Bistorta vivipara*, climate change, ecotypic variation, global warming, plant–soil interactions

## Abstract

Predicting how plants will respond to global warming necessitates understanding of local plant adaptation to temperature. Temperature may exert selective effects on plants directly, and also indirectly through environmental factors that covary with temperature, notably soil properties. However, studies on the interactive effects of temperature and soil properties on plant adaptation are rare, and the role of abiotic versus biotic soil properties in plant adaptation to temperature remains untested. We performed two growth chamber experiments using soils and *Bistorta vivipara* bulbil ecotypes from a subarctic elevational gradient (temperature range: ±3^°^C) in northern Sweden to disentangle effects of local ecotype, temperature, and biotic and abiotic properties of soil origin on plant growth. We found partial evidence for local adaption to temperature. Although soil origin affected plant growth, we did not find support for local adaptation to either abiotic or biotic soil properties, and there were no interactive effects of soil origin with ecotype or temperature. Our results indicate that ecotypic variation can be an important driver of plant responses to the direct effects of increasing temperature, while responses to covariation in soil properties are of a phenotypic, rather than adaptive, nature.

## 2. Introduction

Local adaptation of plants to environmental conditions is receiving increasing interest, particularly in the context of climatic change [1,2]. Patterns of local adaptation provide important insights into how plants might respond to shifts in climatic conditions such as global warming. Therefore, many studies have focused on adaptation of plants to temperature. For example, it has been shown that along large-scale latitudinal temperature gradients, plant ecotypes performed best at their home sites [3,4]. However, temperature does not only directly impact on plant performance, and its effects on other ecosystem components may also indirectly affect performance, thereby modifying local plant adaptation. Previous studies have indicated that plant adaptation to temperature may be coupled with adaptation of aboveground plant-associated organisms to temperature. For example, Laine [5] showed that the relative performance of sympatric versus allopatric populations of fungal pathogens on *Plantago lanceolata* shifted with temperature. Other studies have suggested that plant adaptation to temperature may trade-off against adaptation to insect herbivory. Notably, plants growing at higher elevation would need to allocate fewer resources to herbivore defence because the lower temperatures would moderate herbivore pressure [6].

Few studies, however, have considered interactions with belowground factors [4,7] and we do not know the role of abiotic versus biotic soil properties in determining plant adaptation to temperature. This is despite increased recognition that plant–soil interactions and local adaptation between plants and soil biota can regulate plant growth and fitness [8,9]. Understanding co-adaptation of plants to soil properties and temperature is also important for predicting the potential for evolutionary adaptation to future climatic conditions. Soils generally respond relatively slowly to shifting temperatures [10]; hence, strong local adaptation to soil conditions may impede the ability of plants to track climatic changes (such as those resulting from global warming) which could in turn negatively affect population fitness [11].

We used an elevational gradient in subarctic Sweden to test local plant adaptation to temperature and soil properties. Previous studies in this system have shown that an elevational increase of approximately 500 m corresponds with a decrease in growing season temperature of about 3^°^C, which makes this system powerful for comparisons with predicted future climate change scenarios [12]. Along this gradient, increasing elevation, and thus declining temperature, causes shifts in abiotic and biotic soil properties [13], which can result from direct effects of temperature or from indirect effects through shifts in plant physiology and community composition [14], with potential consequences for plant performance. In particular, it has been shown that rates of mineralization of soil nutrients, and hence availability of nutrients for plant uptake, generally decline with elevation [13,15]. To further our understanding of plant adaptive responses to temperature, it is therefore imperative to consider interactions with both abiotic and biotic soil properties.

We tested patterns of local adaption for *Bistorta vivipara*, a viviparous, perennial, bulbil-producing plant species commonly found in (sub)arctic and alpine habitats [16]. Soils and *B. vivipara* bulbils were collected from three elevations (i.e. three temperature habitats) and seedling responses were tested in two fully reciprocal experiments (with ecotype, temperature and soil origin as factors), using climate-controlled growth chambers to mimic conditions of each temperature habitat. We tested the following hypothesis: local adaptation of *B. vivipara* to temperature is coupled with adaptation to associated soil properties, due in part to co-adaptation of plants and soil organisms. To diagnose local adaptation, we used two non-independent criteria [1,2]: (i) the ‘local versus foreign contrast’ (comparing performance of focal versus other ecotypes in a focal habitat), and (ii) the ‘sympatric versus allopatric contrast’ (comparing performance of a focal ecotype in focal versus other habitats).

## 3. Material and methods

### 3.1 Study site

**Figure 1.**
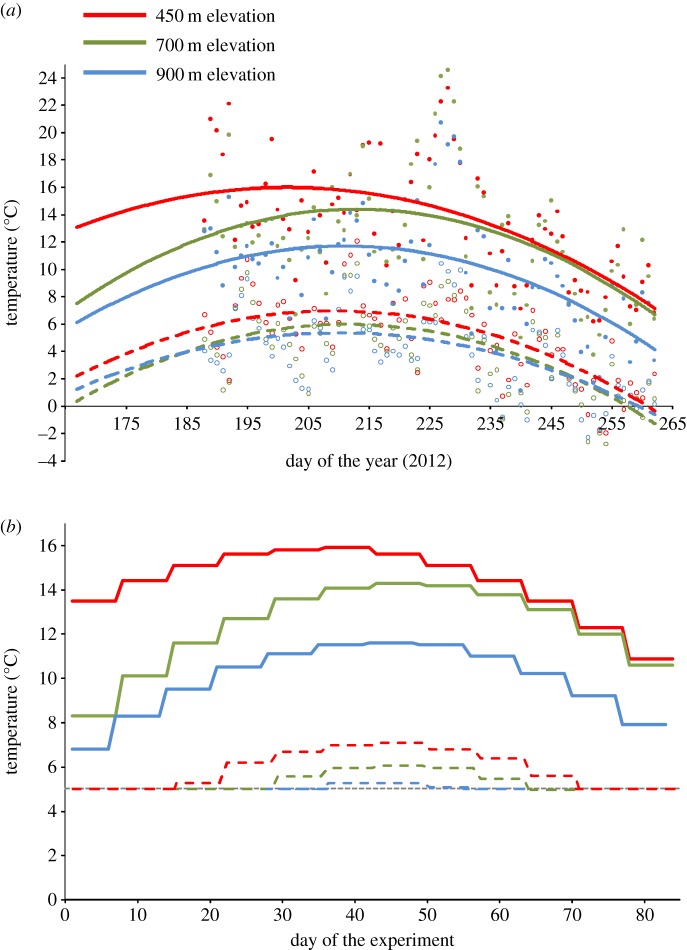
(*a*) Minimum and maximum daily air temperature from early July to mid-September 2012 at 450, 700 and 900 m along the elevational gradient used for our study in Abisko, Sweden. Closed points represent daily maximum temperature. Open points represent daily minimum temperature. Closed and dotted lines show trend lines from quadratic regressions for daily maximum and minimum temperatures, respectively. (*b*) Temperature settings for climate chambers used for testing seedling responses mimicking the temperature conditions at 450, 700 and 900 m as observed along the elevational gradient. Weekly average maximum (closed lines) and minimum temperature values (dotted lines) were derived from equations of the trend lines of regression as shown in (*a*) with the exception that the minimum temperature could not be lower than 5^°^C because of constraints of the growth chambers used (grey dotted line; see Material and methods). Diurnal temperature cycles were set by hourly increments between minimum and maximum values (not depicted in the figure).

Soils and *B*. *vivipara* (syn: *Polygonum viviparum*) bulbil ecotypes used in this study were collected from an elevational gradient on the northeast facing slope of Mount Suorooaivi (1193 m), approximately 20 km southeast of Abisko, Sweden (68^°^21^′^ N, 18^°^49^′^ E) [13,15]. The climate is subarctic with long, cold winters and a short growing season from June to September. The average maximum temperature at 450, 700 and 900 m during the 2012 growing season was 13.5^°^C, 12.3^°^C and 9.7^°^C, respectively; the average minimum temperature corresponded to 5.0^°^C, 4.1^°^C and 3.8^°^C (figure 1*a*). Mean annual precipitation is approximately 310 mm (1913–2000, Abisko Scientific Research Station). Parent soil material is composed of salic igneous rocks and quartic and phyllitic hard schists. The vegetation typically consists of mosaics of two co-dominant types: meadow and heath, which occur across the gradient. Meadow vegetation is diverse and dominated by forbs, graminoids and sedges, while heath vegetation is less diverse and dominated by deciduous and evergreen dwarf-shrubs and *Betula nana*. *Bistorta vivipara* typically occurs in meadow vegetation [13].

### 3.2 Soil collection

Soils for our experiments were collected in August 2012 at 450, 700 and 900 m elevations. Soils were collected to 10 cm depth adjacent to five existing 1×1 m meadow plots at each elevation established earlier in 2012. The mean distance between plots within elevations was *ca* 48 m with the maximum distance between the two plots furthest apart being *ca* 140 m. Because of high spatial heterogeneity over short distances in micro-topography, hydrology and soil fertility in these plant communities [17], it is expected that this distance among plots is sufficient to ensure adequate independence among them [18]. After collection, the soils were frozen (−18^°^C) for six weeks until they were further processed for our experiments; these freezing conditions are well within the range of what these soils experience during the winter.

### 3.3 Soil abiotic and biotic properties

In July 2012, we collected three to six soil cores from each of the five 1×1 m meadow plots at each elevation (45 mm diameter, 10 cm depth) for analysis of abiotic and biotic soil properties. Cores were bulked within plots and kept at 4^°^C overnight before being passed through a 4 mm mesh sieve to remove plant matter and stones. Soil pH was determined on a subsample of fresh soil (2.5 g dry weight) after shaking for 12 h in 40 ml deionized water. Gravimetric moisture content was determined after drying (105^°^C, 24 h) and soil organic matter content was determined after combustion in a muffle furnace (550^°^C, 4 h). A subsample of soil was dried (60^°^C, 72 h), ground and analysed for total carbon (C) and nitrogen (N) by dry combustion using a FLASH 2000 Organic Elemental Analyzer (Interscience, Breda, The Netherlands) and phosphorus (P) with nitric–perchloric acid digestion analysed by inductively coupled plasma [19]. For each subsample of soil, microbial communities were characterized by phospholipid fatty acid (PLFA) analysis [20,21] with different PLFAs representative of different groups of soil microorganisms. We extracted PLFAs from each subsample after freeze-drying and grinding, according to Frostegård *et al.* [22]. We used i14 : 0, 14 : 0, i15 : 0, a15 : 0, 15 : 0, i16 : 0, 16 : 1*ω*9, 16 : 1*ω*7c, 16 : 1*ω*7t, i17 : 0, a17 : 0, 17 : 1*ω*8, cy17 : 0, 17 : 0, 18 : 1*ω*7, cy19 : 0 as indicators for bacteria and 18 : 2*ω*6 as an indicator for fungi.

### 3.4 *Bistorta* ecotypes

*Bistorta vivipara* is a viviparous perennial knotweed commonly found in (sub)arctic and alpine habitats [16,23–25]. Vivipary has been suggested as an adaptation to cold climates [26,27], and in the subarctic tundra of northern Sweden viviparous inflorescences are very common, while the number of flowers is generally low. Occasional sexual reproduction does occur [16,28], but *B. vivipara* normally reproduces asexually by bulbils (vegetative reproduction organs), and this should serve to limit gene flow. Notably, the potential for local adaptation is highest for ‘intermediate’ levels of gene flow [29,30]. *Bistorta vivipara* clones can be widespread [25], but several studies have shown that genotypic variation within and across *B. vivipara*populations in arctic and alpine zones is rather high, with many clones being unique to single populations [16,31,32]. As such, we considered *B. vivipara* a good candidate species to address our hypothesis. At each elevation, *B. vivipara* bulbils were collected within 25 m of the plots. Bulbils were collected between mid-August and early September 2012 from approximately 200 individuals per elevation. These bulbils were stored at 4^°^C for a maximum of six weeks, after which they were surface-sterilized (1% sodium hypo-chloride solution for 1 min) [33], and germinated on sterile soil under common conditions in a greenhouse for use in our growth chamber experiments. Additionally, for each elevation, 50 randomly selected bulbils were oven-dried and weighed in order to compare dry weight among ecotypes.

### 3.5 Experimental design

The soils and bulbil ecotypes were then used in two growth chamber experiments. Experiment 1 addressed whether local adaptation of *B. vivipara* to temperature is coupled with adaptation to associated soil properties. This experiment included three fully crossed factors: soil origin (from 450, 700 and 900 m elevations), bulbil ecotype (from 450, 700 and 900 m elevations) and temperature (mimicking regimes at 450, 700 and 900 m elevations), with five replicates of each treatment combination (3×3×3×5 replicates=135 experimental units). Soils were processed by removing large stones and roots and then homogenized per elevation, because we were interested in local adaptation to soil properties related to elevation, rather than small-scale within-site heterogeneity [34]. For each treatment combination, plastic pots (8×8×10 cm deep, each containing a drainage layer of 150 ml sterilized sand) were filled with the field soil and planted with two pre-germinated *B. vivipara* seedlings.

For each soil origin × ecotype combination, five replicate pots were placed in each of three growth chambers, whose conditions were set to mimic temperature conditions as observed at 450, 700 and 900 m along the elevational gradient during the 2012 growing season (figure 1). Average minimum and maximum temperatures over this period were 5.0 and 13.5^°^C for the 450 m elevation, 4.1 and 12.3^°^C for the 700 m elevation, and 3.8 and 9.7^°^C for the 900 m elevation. Weekly average maximum and minimum temperatures were derived from equations of the polynomial trend lines of regressions as shown in figure 1*a*. Diurnal temperature cycles in the growth chambers were set by hourly increments between minimum and maximum values. Minimum temperature values lower than 5^°^C were set to 5^°^C owing to climate chamber system constraints. Relative humidity was set to 90% in all chambers, and photosynthetically active radiation (PAR) and hours of daylight were set in each growth chamber to represent conditions measured from mid-June to mid-September 2012 in the vicinity of the elevational gradient used in our study (using data derived from the Abisko Scientific Research Station for PAR and from the Swedish Meteorological and Hydrological Institute for day length; electronic supplementary material, figure S1). Each of the five replicates of each treatment combination was randomly assigned to a position in a block within each chamber. All pots were isolated in individual saucers to prevent cross-contamination through water movement. Pots were rotated systematically between chambers (i.e. entire temperature treatments moved between chambers) on a weekly basis to minimize effects of any uncontrolled sources of variation among chambers. Pots were watered as necessary throughout the experiment to eliminate moisture as a limiting factor.

Experiment 2 addressed whether local adaptation to temperature is due in part to co-adaptation of plants and soil organisms; it differentiated between the effects of abiotic and biotic soil properties using sterilized soils and living soil inocula. By comparing sterilized soils to reinoculated soils, the effects of abiotic versus biotic soil factors on plant growth can be effectively separated [33,35]. The experiment followed a full-factorial design including inoculum origin (inoculum from 450, 700 and 900 m elevations, and a non-inoculated sterile control), bulbil ecotype (from 450, 700 and 900 m elevations) and temperature (mimicking regimes at 450, 700 and 900 m elevations), with five replicates of each treatment combination (4×3×3×5 replicates = 180 experimental units). Soils were processed as described for experiment 1. We then took an equal subsample of soil from each of the three elevations and combined them into one mixed bulk soil which we sent to Synergy Health (Swindon, UK) for *γ*-irradiation (25 kGy), which is widely used for killing soil biota [33]. We prepared four inoculation treatments, i.e. a ‘control’ sterilized soil without any inoculation, or inoculation of the sterilized soil with live soil from each of the three elevations. For each of the three treatments in which inoculation was performed, a volume of 10% live soil from one of the three elevations was mixed with the bulked sterilized soil; for the ‘control’ soil only bulked sterilized soil was used. The soil inocula potentially included all soil microflora (prokaryotes and fungi), and micro- and mesofauna. The inoculated soils were then placed in pots and planted with *B. vivipara* seedlings as described for experiment 1. The experiment was then run following approaches and methodology described for experiment 1.

For both experiments, plants were grown for 12 weeks, following temperature and light conditions characteristic for the Abisko growing season (figure 1; electronic supplementary material, figure S1). Experiments like this are usually run for the duration of several months [33,36], and typically focus on the seedling stage because seedling performance is an important component of plant fitness [37]. At harvest, soil was carefully washed from the roots and roots and shoots were separated. Shoot and root dry mass were determined after drying at 60^°^C for at least 72 h.

### 3.6 Data analyses

The effects of ecotype, temperature and soil/inoculum origin, and their interactions, on total plant biomass were tested using three-way ANOVA. Biomass of the two seedlings per pot was averaged, and pots served as the unit of replication (*n*=5). To assess significant differences among means, Tukey's HSD *post hoc* tests with Bonferroni corrections (*p*=0.05) were run for (i) ‘local versus foreign’ comparisons, and (ii) ‘sympatric versus allopatric’ comparisons [1], i.e. pairwise comparison of the levels of each factor within the levels of the other factor in the interaction [38]. Effects of ecotype on bulbil dry weight and effects of elevation on abiotic and biotic soil properties were tested using one-way ANOVA with Tukey's HSD *post hoc* tests. For all data analyses, data were transformed when necessary to meet the assumptions for parametric testing. All statistical analyses were performed in SPSS (PASW statistics v. 21.0, IBM Corporation, Armonk, NY, USA).

## 4. Results

### 4.1 Experiment 1

**Figure 2.**
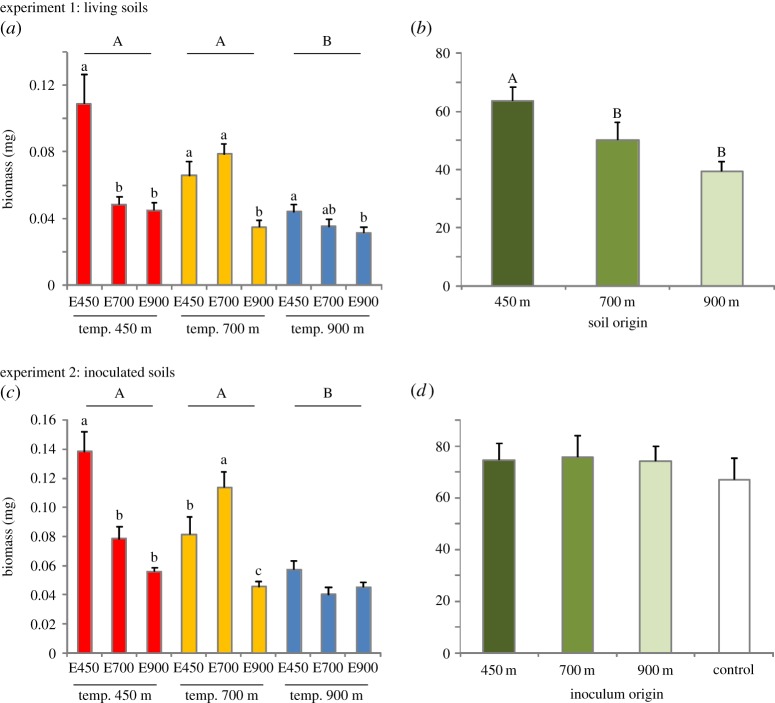
Biomass responses (mean ± s.e., *n*=5) of *Bistorta vivipara* ecotypes under temperature and soil treatments (local versus foreign contrast). Bulbil ecotypes were collected from 450 m (E450), 700 m (E700) and 900 m (E900) elevations and grown under temperature regimes (‘temp’) associated with 450, 700 and 900 m elevations. (*a*) Biomass responses in living soils under temperature regimes associated with 450, 700 and 900 m elevations. (*b*) Biomass responses in living soils collected from 450, 700 and 900 m elevations, averaged across all temperatures. (*c*) Biomass responses in sterilized soils re-inoculated with living soil inoculum under temperature regimes associated with 450, 700 and 900 m elevations. (*d*) Biomass responses in sterilized soils re-inoculated with living soil inoculum collected from 450, 700 and 900 m elevations, and in sterilized soil without inoculum (control), averaged across all temperatures. Different capital letters denote significant differences (Tukey's HSD *post hoc* tests, *p*<0.05) among ecotypes (*a*,*c*) or soil origins (*b*). Different lower case letters denote significant differences among temperature treatments within ecotypes.

There was a significant interactive effect between ecotype and temperature on plant biomass, but there were no interactions of soil origin with ecotype or temperature (table 1). Furthermore, plant biomass was higher in the 450 m soil than the 700 and 900 m soils (figure 2*b*). Local versus foreign contrast (figure 2*a*): at the 450 m temperature, biomass of the 450 m ecotype was higher than for the 700 and 900 m ecotypes; at the 700 m temperature, biomass of the 450 and 700 m ecotypes was higher than the 900 m ecotype; and at the 900 m temperature, biomass of the 450 m ecotype was higher than the 900 m ecotype. Sympatric versus allopatric contrast (electronic supplementary material, figure S2*a*): for the 450 and 700 m ecotypes, biomass was highest at temperature treatments corresponding to their elevation of origin; and there was no temperature effect on the ecotype from 900 m.

**Table 1.** Results from ANOVA of effects of ecotype (450, 700, 900 m), temperature regime (450, 700, 900 m) and soil or inoculum origin (450, 700, 900 m; control (experiment 2 only)) on total biomass of *Bistorta vivipara* grown on living and inoculated soils. (Significant *p*-values (<0.05) are in bold type. Non-significant two- and three-way interactions are not shown.)

	living soils			inoculated soils		
effect	DF_num_	*F*-value	*p*-value	DF_num_	*F*-value	*p*-value
ecotype (*E*)	2	31.78	**<0.001**	2	20.64	**<0.001**
temperature (*T*)	2	25.75	**<0.001**	2	21.55	**<0.001**
soil origin (*S*)	2	21.07	**<0.001**			
inoculum origin (*I*)				3	0.50	0.69
*E*×*T*	4	9.20	**<0.001**	6	11.22	**<0.001**
error	108			144

### 4.2 Experiment 2

Plant biomass patterns in experiment 2 were generally similar to those observed in experiment 1. Local versus foreign contrast (figure 2*c*): at the 450 m temperature, the 450 m ecotype had significantly higher biomass than did the 700 and 900 m ecotypes; similarly, at the 700 m temperature, the 700 m ecotype had a significantly higher biomass than did the two other ecotypes; and at the 900 m temperature there were no significant biomass differences between the three ecotypes (figure 2*b*). Plant biomass was not affected by inoculum origin, or by its interaction with ecotype and/or temperature (figure 2*d*). Sympatric versus allopatric contrast (electronic supplementary material, figure S2*b*): for the 450 and 700 m ecotypes, biomass was highest at temperature treatments corresponding to their elevation of origin; and there was no temperature effect on the ecotype from 900 m. Biomass of the 450 and 700 m ecotypes differed significantly from that of the 900 m ecotype, but not from each other.

## 5. Discussion

**Figure 3.**
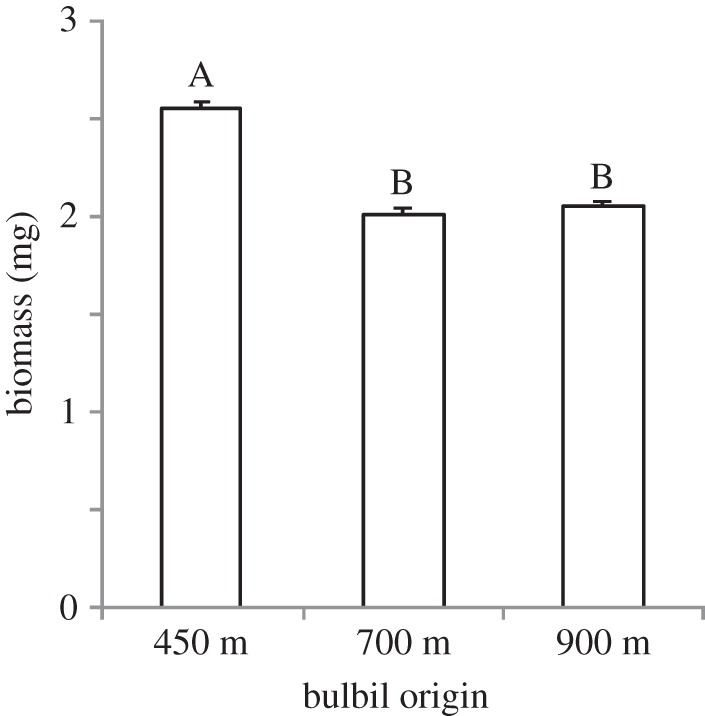
Biomass (mean ± s.e., *n*=50) of bulbils of *Bistorta vivipara* ecotypes collected from 450, 700 and 900 m elevations. Bulbil biomass was significantly affected by elevation (ANOVA: *F*_2,147_=86.47, *p*<0.001). Different capital letters denote significant differences (Tukey's HSD *post hoc* tests, *p*<0.05) among elevations.

Disentangling the importance of covarying factors enhances our understanding of plant adaptation to local environmental conditions. We found partial evidence for local adaptation of *B*. *vivipara* to shifting conditions with elevation, largely manifested by adaptation to temperature. Ecotypes from the lowest elevation performed best at local temperatures (i.e. sympatric versus allopatric contrast), and at temperatures representing low elevation the local ecotype performed best (i.e. local versus foreign contrast). Similar patterns were found for the mid-elevation ecotype and temperatures. These patterns could not solely be explained by the more favourable temperature conditions at lower elevations (i.e. intrinsic differences in habitat quality [2]). Notably, mid-elevation ecotypes did better at mid-elevation temperature which is on average 1.4^°^C lower than low elevation temperature; this is despite observations that growth of *B. vivipara* in the subarctic is temperature limited [23,24,39]. Moreover, while maternal effects could have contributed to enhanced performance of local ecotypes at low elevation (i.e. bulbils from 450 m were larger than bulbils from other elevations; figure 3), these effects could not account for the ‘local versus foreign’ comparison at mid elevation. In this light, ecotypes from mid elevation performed better at mid-elevation temperatures than at more favourable low-elevation temperatures. Taken together, although we do not know the level of clonal diversity between bulbil ecotypes, the large differences among ecotypes in biomass response to temperature strongly suggest a genetic basis for the observed patterns.

Contrary to our hypothesis, we found that plant adaptation to temperature was independent of soil conditions. Overall, plants performed best in low elevation soils, probably because of increased soil fertility at lower elevations [13,40]. However, contrary to previous studies [9,41,42], we found no evidence of local adaptation to soil abiotic and/or biotic properties. It has been suggested that local adaptation to soil conditions might be particularly important in ‘extreme’ soil types [4], as has been shown, for example, in nutrient-poor serpentine soils [43]. As such, low soil fertility at the highest elevation [13] would be expected to exert a strong selection pressure. However, in subarctic ecosystems, spatio-temporal variation in soil nutrient availability is strong even within elevations [17], which could work against local adaptation to soil conditions [4]. Moreover, plants were unresponsive to soil biota regardless of soil origin. Strong environmental control (i.e. temperature, soil abiotic properties) of plant growth may have suppressed effects of soil biota, or effects of mutualists and pathogens may have cancelled each other out.

Understanding local adaptation of plants to environmental conditions is important in predicting plant performance and population dynamics under future climate change scenarios [44]. Our results show that with shifts in elevation, plants adapt to local environmental conditions, but that adaptation was driven solely by responses to variation in temperature. Plant growth was responsive to variation in associated soil properties, but these responses were decoupled from local adaptation to temperature. As such, our results indicate that the direct effects of global warming on plant performance depend on the responsiveness of ecotypic variation to temperature, while plant responses to temperature-induced shifts in soil properties may be primarily phenotypic. The decoupling of plant responses to temperature and soil properties that we observed also suggest that the potential for adaptation of *B. vivipara* to changing future temperatures would largely depend on the genetic variability in temperature optimum, and would not be impeded by soil legacies. As such, our study sheds new light on the relative importance of genetic adaptation and phenotypic plasticity for plant performance under shifting climatic conditions.

## Supplementary Material

Titlle: Supporting data Description: Table showing soil properties for soils collected along the elevational gradient used for our study. Figure showing photosynthetically active radiation (PAR) and day length values. Figure showing biomass responses of Bistorta vivipara ecotypes under temperature and soil treatments (sympatric vs. allopatric contrast).

## Data accessibility

The datasets supporting this article have been deposited in the Dryad repository [45]: doi:10.5061/dryad.v2k50.
