# Supplementary material for: Local plant adaptation across a subarctic elevational gradient
Source: R Soc Open Sci. 2014 Nov 12;1(3):140141. doi: 10.1098/rsos.140141 (PMC4448849; doi:10.1098/rsos.140141)
Supplement: Titlle: Supporting data Description: Table showing soil properties for soils collected along the elevational gradient used for our study. Figure showing photosynthetically active radiation (PAR) and day length values. Figure showing biomass responses of Bistorta vivipara ecotypes under temperature a [file rsos140141supp1.docx]

**Electronic supplementary material – Table**

Table S1. Soil properties (mean ± s.e., N = 5; and F- and *P*-values from one way ANOVA) in the 0-10 cm depth soil layer for soils collected at 450 m, 700 m, and 900 m along the elevational gradient used for our study in Abisko, Sweden. Significant *P*-values (*p* ≤ 0.05) are bolded. Different letters denote significant differences among elevations (Tukey post hoc tests, *P* < 0.05).

| Variable | 450 m | 700 m | 900 m | F_2,8_ | *P* |
| --- | --- | --- | --- | --- | --- |
| pH | 5.5 ± 0.1^b^ | 5.6 ± 0.1^b^ | 6.1 ± 0.1^a^ | 9.72 | **0.003** |
| Soil organic matter (%)^1^ | 9. 5 ± 1.8^b^ | 21.1 ± 3.9^a^ | 13.8 ± 1.7^ab^ | 4.88 | **0.028** |
| C:N ratio^b^ | 16.9 ± 1.4 | 17.0 ± 2.1 | 14.7 ± 1.4 | 0.69 | 0.520 |
| C:P ratio^b^ | 147.2 ± 4.8 | 146.8 ± 18.3 | 150.6 ± 28.9 | 0.02 | 0.983 |
| N:P ratio | 9.0 ± 0.9 | 8.8 ± 0.8 | 10.0 ± 1.3 | 0.35 | 0.662 |
| Fungal PLFA^2^ (nmol g^-1^ organic matter)^1^ | 210.3 ± 23.5^a^ | 275.4 ± 40.8^a^ | 103.3 ± 13.9^b^ | 16.74 | **0.001** |
| Bacterial PLFA (nmol g^-1^ organic matter) | 1957.6 ± 110.8^a^ | 1682.7 ± 214.7^ab^ | 1235.2 ± 147.5^b^ | 13.80 | **0.003** |
| Fungal:bacterial PLFA ratio^a^ | 0.10 ± 0.01^a^ | 0.16 ± 0.01^b^ | 0.10 ± 0.10^a^ | 15.28 | **0.002** |

^1^Data ln(x)-transformed before analysis

^2^Phospholipid fatty acid

**Electronic supplementary material – Figures**

**Figure legends**

Figure S1. A) Daily averaged photosynthetically active radiation (PAR) (left axis) and day length values (right axis) from mid June to mid September 2012 (data derived from the Abisko Scientific Research Station (Abisko, Sweden) and the Swedish Meteorological and Hydrological Institute, respectively). B) Daily PAR (left axis) and day length averages (right axis) used to program chambers for testing seedling responses mimicking field conditions along the elevational gradient.

Figure S2. Biomass responses (mean ± s.e., N=5) of *Bistorta vivipara* ecotypes under temperature and soil treatments: ‘sympatric vs. allopatric contrast’. Bulbil ecotypes were collected from 450 m, 700 m and 900 m elevations and grown under temperature regimes associated with 450 m (T450), 700 m (T700), and 900 m (T900) elevations. Experiment 1: A) Biomass responses in living soils under temperature regimes associated with 450 m (T450), 700 m (T700), and 900 m (T900) elevations. Experiment 2: B) Biomass responses in sterilized soils re-inoculated with living soil inoculum under temperature regimes associated with 450, 700, and 900 m elevations. Different capital letters denote significant differences (Tukey post hoc tests, *P* < 0.05) among ecotypes. Different lower case letters denote significant differences among ecotypes within temperature treatments.

Figure S1

Figure S2
